# Investigation of Electromagnetic Wave Propagation in Photonic-like Welded Materials Using the Finite Element Method

**DOI:** 10.3390/ma17225498

**Published:** 2024-11-11

**Authors:** Ayse Nihan Basmaci, Seckin Filiz

**Affiliations:** Vocational School of Technical Sciences, Tekirdag Namik Kemal University, 59030 Tekirdag, Turkey; sfiliz@nku.edu.tr

**Keywords:** electromagnetic wave propagation, finite element analysis, material property parameters, photonic crystals, rotary inertia welding

## Abstract

In this study, two identical and two dissimilar materials are conjoined by applying the friction welding method to yield various rods. This investigation’s primary focus entails examining the repercussions associated with the heat-affected zone (HAZ) arising from elevated temperatures at the welding interfaces on the propagation of electromagnetic (EM) waves within the resultant structures. The study incorporates the photonic crystal approach in conjunction with Maxwell’s equations, and the subsequent solution of the latter is executed using the finite element method. The subdivision of the structures into fifteen elements is predicated upon the assumption of the electromagnetic wave number of the m-th segment, k_m_, of discrete segments. The finite element method is then administered to the HAZ regions of the structures, wherein the HAZ is discretised into one, three, and five elements, respectively.

## 1. Introduction

Owing to the rapid advances in telecommunication and satellite technologies, photonic structures are becoming increasingly important [1,2,3]. Photonic crystal structures are generally artificially produced periodic segments in one dimension [4,5,6], which manipulate the propagation of electromagnetic waves [7,8]. In two-dimensional and three-dimensional structures, electromagnetic waves exit the photonic structure by following a specific path [9,10,11,12]. Similarly, travelling waves may have an ultrasonic structure rather than an electromagnetic one, which has been the subject of some studies [13,14,15]. These structures, which use ultrasonic and acoustic properties, are known as phononic crystals [16,17,18,19]. Whether examining electromagnetic or mechanical (acoustic) waves, the important issue is that the sum of the travelling wave and the reflected waves is equal to the incident wave [20]. This property has been used to study the characteristics of the reflected and travelling waves in the structures [21,22,23,24,25]. Especially in recent years, thanks to developments in material technology, the absorption of electromagnetic waves emitted from radars is realised, which is a concept known as stealthy [26,27,28]. There are also studies in the literature in which the electromagnetic wave propagation equation is derived from Maxwell’s equations [29,30]. The finite element method is a comprehensive approach widely employed to address diverse engineering challenges [31,32]. In addition, the finite element method allows for studying electromagnetic (EM) wave propagation in complex structures. Therefore, crack propagation [33], mechanical wave propagation in elastic bars [34,35], seismic wave propagation in various structures [36], and the properties of electromagnetic wave propagation in ordered photonic crystals can also be studied [37].

This study undertakes a theoretical exploration of electromagnetic wave propagation behaviour in experimentally fabricated welded structures. These structures are assembled by welding two identical or different materials, resulting in joint zones exposed to elevated temperatures [38,39,40,41]. Unlike a prior study [41] focusing solely on the mechanical properties (modulus of elasticity) of these structures and forecasting a mechanical functional grading, this study takes a distinctive direction. It investigates the potential of joining two dissimilar structures via inertia friction welding, considering the limitations of traditional welding methods. During the formation of these structures, heat-affected zones (HAZs) develop in the joint zones, representing transition zones. It is essential to recognise that the finite element method, finite difference method, and method of moments (MoM) are the principal techniques applied in analysing metamaterials and photonic materials to address problems related to electromagnetic radiation. This study employs the finite element method to distinguish between the heat-affected zones (HAZs) and the heat-unaffected regions of photonic-like samples manufactured through frictional welding. This methodology facilitates a detailed examination of the electromagnetic wave propagation behaviour within each element representing the HAZs and rods. As such, the focus on analysing electromagnetic wave propagation, particularly within the heat-affected HAZ regions, combined with applying a photonic-like structure approach, distinguishes this study from other research in the current literature [42,43,44].

Consequently, the resultant structures comprise three distinct components: the first metal, the intermediate region (HAZ), and the second metal. What distinguishes this study is its specific examination of the electromagnetic wave propagation properties in the HAZs of the produced materials. The study involves transferring alterations in the material property parameters of the structures to the wave equation utilising the finite element method to ascertain the electromagnetic wave propagation behaviour in the HAZs. This procedure entails incremental modelling of the structures in each element. While the investigations on the HAZ regions are being conducted, the changes occurring in the HAZ regions are modelled with the change occurring in the electromagnetic material property parameters. The relevant equations are created in light of this modelling. The variations in the electromagnetic material properties associated with the heat-affected zones, or transition zones, are represented by the symbols I, v, and n within the framework of modelling based on the finite element method.

In this study, a detailed investigation into the propagation behaviour of electromagnetic waves in various samples, independent of any external pulses or electromagnetic forces, is conducted. The analysis focuses on the relationship between this behaviour and changes in material property parameters, specifically permeability and permittivity.

## 2. Materials and Methods

### 2.1. Experimental Setup and Manufacturing of Friction Welded Rods

**Figure 1:**
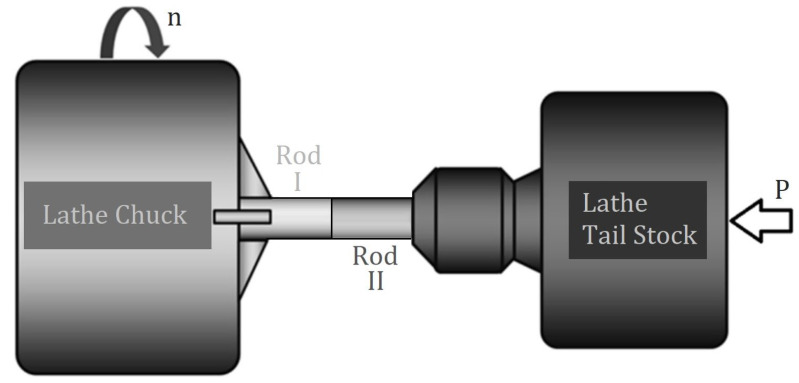
Rotary inertia friction welding using a universal lathe machine.

Rods are created using rotary inertia friction welding. Unlike traditional welding, which only joins similar materials, rotary inertia welding can join different materials. As shown in Figure 1, two rods attached to a universal lathe undergo rotary inertia welding by generating friction through the lathe chuck’s speed (n) and the pressure (P) from the lathe tailstock.

**Figure 2:**
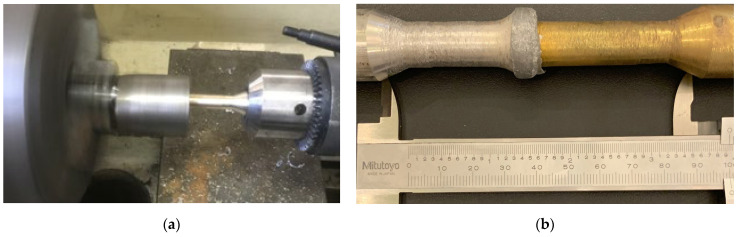
(**a**) Experimental setup of the rods of the different types (S2 type) produced by inertia friction welding; (**b**) welded rods (S1 type).

**Figure 3:**
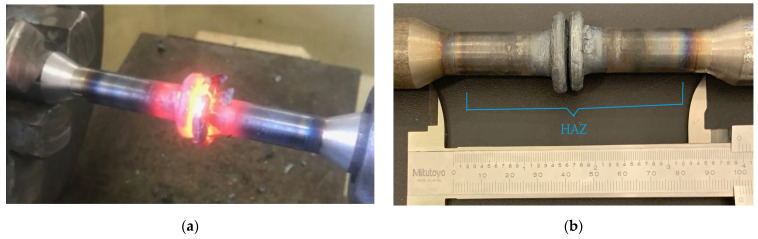
(**a**) Rods of the S2 type produced by inertia friction welding; (**b**) illustration of the colour spectrum change in the HAZ in the S2 type.

Figure 2 and Figure 3 depict the S1 sample, created experimentally from different rods, and the S2 sample, produced from the same rods. In the S1 sample, axial misalignment is possible due to the rapid melting of the rod with a low melting temperature. As shown in Figure 2, it is recommended to apply weight to the weld section to prevent misalignment. The inclusion of weight in the inertia friction welding process, as depicted in Figure 2a, introduces a unique aspect to this study regarding originality. The application of weight enables a more precise and efficient inertia friction welding process. In Figure 2b, the light grey material represents aluminium, the molten metal, while the yellow material depicts brass. The utilisation of weight during the inertia friction welding process facilitates the provision of axial stability during the melting of aluminium towards brass.

Upon examining the sample depicted in Figure 3, two steel materials of identical composition undergo the friction welding process, combining them to create a seamless joint (HAZ). The colour variation reveals the presence of the heat-affected zone (HAZ), encompassing the welding area and its immediate vicinity.

This study delves into the impact of the heat-affected zone (HAZ) generated by elevated temperatures at welding interfaces on the behaviour of electromagnetic (EM) waves within the resulting structures. To accomplish this, the investigation will employ the photonic crystal approach in tandem with Maxwell’s equations. The resulting solutions derived from Maxwell’s equations will be obtained using the finite element method.

### 2.2. Electromagnetic Wave Propagation Approach for Produced Rods

**Figure 4:**
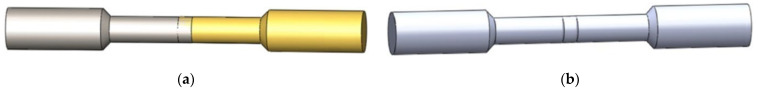
(**a**) Friction-welded dissimilar rods (S1); (**b**) friction-welded similar rods (S2).

The schematic representations of the samples created through friction welding are depicted in Figure 4. Upon examining the behaviour of electromagnetic wave propagation in these samples, it is prudent to initially acknowledge that they comprised two distinct segments. Subsequently, finite element modelling will be conducted in three distinct sections, accounting for variations in the electromagnetic material properties of the heat-affected zone (HAZ) regions in the produced samples.

The produced rods are analysed using a photonic crystal approach to investigate the behaviour of electromagnetic wave propagation using Maxwell’s equations.

In a source-free, linear, isotropic and homogenous region, the first-order Maxwell’s curl equations are [29]

(1a) $$\nabla \cdot \vec{E}=0,$$

(1b) $$\nabla \cdot \vec{H}=0,$$

(1c) $$\nabla \times \vec{E}=-i\omega \mu \vec{H},$$

(1d) $$\nabla \times \vec{H}=-i\omega \varepsilon \vec{E}.$$

where *µ* is the permeability, *ε* is the permittivity, $\vec{E}$ is the electrical field, $\vec{H}$ is the magnetic field and *ω* represents the frequencies associated with the propagation of electromagnetic waves. Using Equations (1c) and (1d), Equation (2) is obtained as follows:

(2) $$\nabla \times \left(\nabla \times \vec{H}\right)=\nabla \left(\nabla \cdot \vec{H}\right)-\nabla^{2}\vec{H}=\nabla \times \left(-\mu \frac{\partial \vec{H}}{\partial t}\right)$$

By solving Equation (2), the partial differential equation (wave equation) depending on time and position is obtained as follows:

(3) $$\frac{\partial^{2}H_{x}}{\partial x^{2}}+\mu \varepsilon \frac{\partial^{2}H_{x}}{\partial t^{2}}=0$$

Accordingly, in a two-segment perfect bonding structure, the displacement functions of electromagnetic waves are defined as $H_{x(1)}=h_{\left(1\right)in}e^{i(\omega t-k_{1}x)}+h_{\left(1\right)r}e^{i(\omega t+k_{1}x)}$ and $H_{x(2)}=h_{\left(2\right)t}e^{i(\omega t-k_{1}x)}$. Herein, $h_{\left(1\right)in}$ represents the incident wave, $h_{\left(1\right)r}$ represents the reflected wave, $h_{\left(2\right)t}$ represents the transmitted wave, $k_{1}$ represents the wave number in the 1-st segment, $k_{2}$ represents the wave number in the (2)nd segment and ω represents the frequency values of wave propagation. Transmitted and incident waves are as follows:

(4a) $${\left(k_{1}\right)}^{2}h_{(1)in}-\mu_{1}\varepsilon_{1}{\left(\omega_{1}\right)}^{2}h_{(1)in}=0,$$

(4b) $${\left(k_{2}\right)}^{2}h_{(2)t}-\mu_{2}\varepsilon_{2}{\left(\omega_{2}\right)}^{2}h_{(2)t}=0.$$

Equations (4a) and (4b) are utilised to derive the wave number as specified in Equation (5):

(5a) $$k_{2}=k_{1}\sqrt{\frac{\mu_{2}\varepsilon_{2}}{\mu_{1}\varepsilon_{1}}}$$

(5b) $${\left(k_{2}\right)}^{2}h_{(2)t}-\mu_{2}\varepsilon_{2}{\left(\omega_{2}\right)}^{2}h_{(2)t}=0.$$

In order to determine the propagating wave, it is imperative to possess knowledge of the associated reflection values [19]. Within this context,

(6a) $$R={\left(\frac{k_{1}-k_{2}}{k_{1}+k_{2}}\right)}^{2},T=\frac{4k_{1}k_{2}}{{\left(k_{1}+k_{2}\right)}^{2}},$$

(6b) T+R=1.

**Figure 5:**
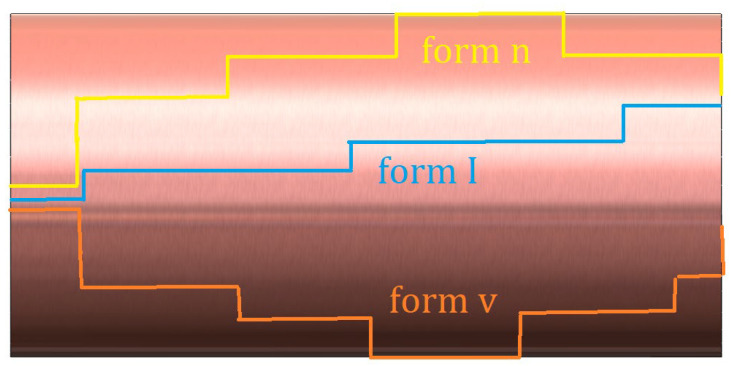
Elemental illustration of the transition zone (HAZ).

The determination of frequency values in photonic structures with isotropic electromagnetic material properties is accurately accomplished using Equations (4a) and (4b). Calculations for the transmission and reflection of travelling waves are performed using Equations (5a), (5b) and (6b). These equations are integral in analysing the behaviour of electromagnetic wave propagation in photonic structures with perfect bonding. It is noteworthy that the structures under investigation are subjected to high temperatures during the welding process, leading to the formation of a transition zone (HAZ) in the joint regions. Consequently, the material property parameters in these transition zone segments are denoted as *μ*_m_*ε*_m_, *μ*_m+1_*ε*_m+1_ and so on, introducing complexities in studying the behaviour of electromagnetic wave propagation in these structures. To overcome this challenge, the finite element method can be applied to adjust the material properties in the transition zones based on the number of elements. The analysis of the electromagnetic material parameter properties in this context is depicted in Figure 5.

In the context of Figure 5, the material property parameter represented by I is depicted as displaying a continuous increase. The parameter represented by n shows an initial increase followed by a decrease, while the parameter represented by v exhibits a continuous decrease, contrasting with the behaviour of the n parameter. The heat-affected zone depicted in Figure 5 is analysed using various element configurations, including five, elements three elements, and one element.

The analysis carried out using the finite element method on the frictionally welded samples is selected because it effectively addresses the electromagnetic wave propagation equation, which is crucial for understanding the behaviour of electromagnetic waves in each specific part of the produced samples. In contrast to the studies referenced in [41,45,46], which examined only the mechanical or thermal properties of the heat-affected zones (HAZs), this study uniquely focuses on analysing the electromagnetic wave propagation behaviour. It achieves this by segmenting the samples into equal-length sections: Part I, Part II, and the HAZ.

**Figure 6:**
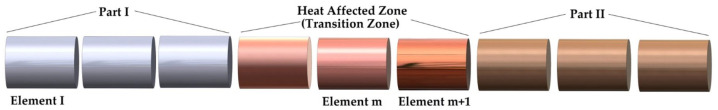
Finite element illustration of a welded sample.

As depicted in Figure 6, the HAZ of the welded structure is subdivided into up to 5 segments and examined in a stepwise manner with a maximum of 15 elements in total. The material parameters within the HAZ, as outlined in Figure 5, are individually modelled for each element, highlighting distinct material properties. These variations in material properties play a significant role in influencing the transmission logic and waveforms of the electromagnetic (EM) wave. The primary objective of this study is to comprehensively investigate and analyse these effects. To this end, the essential analyses are systematically conducted utilising finite difference modelling, as described in Equations (7)–(10), with the ensuing results indicating the feasibility of employing this modelling approach for complex structures.

As shown in Figure 6, the finite element modelling of the m-th element of the structure, which is characterised by a transition zone generated from a source divided into three, is conducted using Equation (3) as outlined below:

(7) $$\frac{\partial^{2}H_{x(m)}}{\partial x^{2}}+{\left(\mu \varepsilon \right)}_{m}\frac{\partial^{2}H_{x(m)}}{\partial t^{2}}=0$$

Upon rearranging Equation (7) to solve for the m-th element, with the displacement function denoted as $H_{x(m)}=h_{\left(m\right)}e^{i(\omega_{m}t-k_{m}x)}$, it becomes feasible to calculate the propagation frequencies of the electromagnetic wave for each individual element. When examining the propagating and reflecting waves, it is imperative to take into account Equations (5a), (5b) and (6b). As such, rearranging Equation (7) for the m-th element,

(8) $$H_{x(m)}-\frac{{\left(\mu \varepsilon \right)}_{m}\omega^{2}}{{k_{m}}^{2}}H_{x(m)}=0$$

$p_{m}=\frac{{\left(\mu \varepsilon \right)}_{m}}{{k_{m}}^{2}}$ is expressed as the electromagnetic material parameter for the m-th element. Thus, Equation (8) becomes as follows in finite element form:

(9) $$K_{m}H_{x(m)}+{\omega_{m}}^{2}M_{m}H_{x(m)}=0$$

Here, $K_{m}=1$ is a constant value, and $M_{m}=p_{m}$ is the material property parameter for the m-th element. Accordingly, the constant eigenvector *K* and the material property parameter eigenvector *M* are expressed as follows:

(10) $$\left[K\right]=\left[\begin{array}{ccc} 1 & -1 & \ldots \\ -1 & 2 & \ldots \\ 0 & \ldots & \ldots \end{array}\right],\left[M\right]=\left[\begin{array}{ccc} p_{1} & -p_{1} & \ldots \\ -p_{1} & p_{1}+p_{2} & \ldots \\ 0 & \ldots & \ldots \end{array}\right]$$

The process of obtaining eigenvalues involves using eigenvectors. Specifically, electromagnetic frequency values, which are known as eigenvalues, can be calculated by solving the equation $\left|\left[K\right]-{\omega_{m}}^{2}\left[M\right]=0\right|$. Equation (10) provides data pertaining to the maximum 15 × 15 matrix solution, which comprises a total of 15 elements organised into groups of five elements each. Additionally, the analysis is conducted using MathCAD 14 (Parametric Technology Corporation, Boston, MA, USA), to facilitate the resolution.

## 3. Results and Discussions

The Mode 1 frequencies are determined by using Equation (10), which is derived using the finite element method. The frequencies for Mode 1 are listed in both Table 1 and Table 2. These frequencies are associated with the propagation of electromagnetic waves in samples S1 and S2 that are created through welding. The Mode 1 frequencies vary depending on the materials used to produce the S1 and S2 samples. The material property parameters for each element of the S1 sample can be found in Table 1, and those for the S2 sample can be found in Table 2. The highest frequency for electromagnetic wave propagation is 1.58 rad/s (0.252 Hz) in the n form, corresponding to the largest material property parameter. The lowest frequency for electromagnetic wave propagation is 0.707 rad/s (0.112 Hz) in the v form when the material property parameter is 0.5. As the S2 sample is created by welding two identical materials together, the I form is not present for this sample. Therefore, the v and n forms are considered for sample S2. The S1 sample is in the v form with the lowest transmission ratio of 0.57. Additionally, the highest transmission ratio is found in the I form for sample S1, while it is found in the n form for sample S2.

Table 2 provides the material property parameters for the S2 sample, comprising 15 elements.

Table 1 shows the distribution of material property parameters for sample S1 according to the elements, while Table 2 shows the distribution of material property parameters for sample S2 according to the elements.

Table 3 presents the error analysis results for the S1 sample. The data indicate that the highest recorded error margin is 4.396%, which reflects a relatively low level of error. Notably, even at this level of error, the analysis tables for three elements are deemed usable. Furthermore, the error margin decreases to 2.697% when five elements are considered.

The error analysis results for the S2 sample are presented in Table 4. As shown, the highest error margin recorded is 0.701%, which indicates that it remains at a very low level. Even under these circumstances, the approximation tables created for three elements can be efficiently utilised.

**Figure 7:**
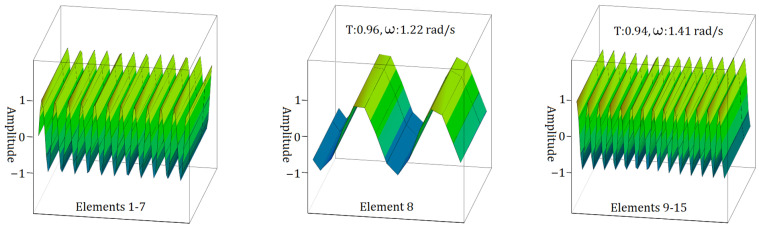
Electromagnetic (EM) wave propagation behaviour in sample S1 with a single element in the transition zone for the case of I form.

**Figure 8:**
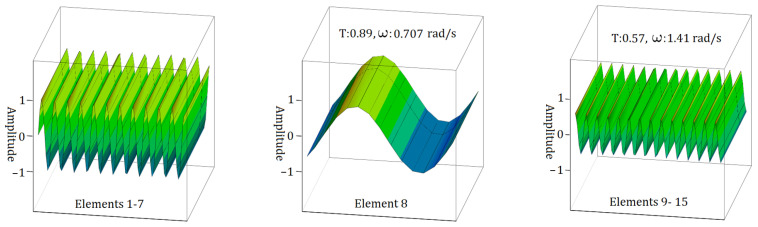
EM wave propagation behaviour in sample S1 with a single element in the transition zone for the case of v form.

**Figure 9:**
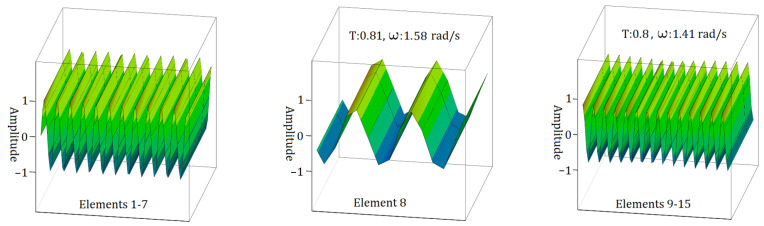
EM wave propagation behaviour in sample S1 with a single element in the transition zone for the case of n form.

Figure 7, Figure 8 and Figure 9 depict the behaviour of electromagnetic wave propagation in sample S1. Additionally, the transmission of the propagating wave is visible in every element of sample S1. In Figure 7, it is evident that for the case of the I form of the S1 sample transition zone, the maximum frequency of electromagnetic wave propagation is recorded at 1.41 rad/s for elements within the range of 9 to 15. Concurrently, the transmission ratio (T) exhibits a gradual decline, initially decreasing to 0.96 and subsequently to 0.94.

In Figure 8, regarding the v form of the S1 sample transition zone, the highest electromagnetic frequency value is observed for the case between Elements 9 and 15, with ω measuring 1.41 rad/s, as shown in Figure 7. Meanwhile, the transmission ratio gradually decreases, dropping to 0.89 and then to 0.57, indicating a significant reduction in the transmission ratio. Additionally, the frequency values for electromagnetic wave propagation, derived from the analysis of the Element 8 state, are notably low, with ω at 0.707 rad/s. This lowest frequency value of ω = 0.707 rad/s for the case involving eight elements is attributed to the direct proportionality between the material property parameters and the frequencies of electromagnetic wave propagation.

In Figure 9, the n form of the S1 sample transition zone shows that the highest frequency for electromagnetic wave propagation occurs with Element 8, which has a value of ω = 1.58 rad/s, as illustrated in Figure 7. The transmission ratios (T) remain consistent, showing slight variation, especially between Element 8 and Elements 9–15, with values ranging from 0.81 to 0.80.

**Figure 10:**
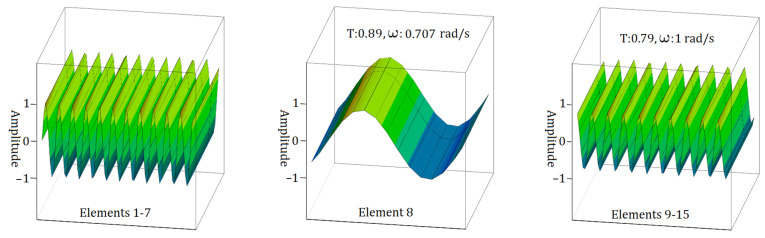
EM wave propagation behaviour in sample S2 with a single element in the transition zone for the case of v form.

**Figure 11:**
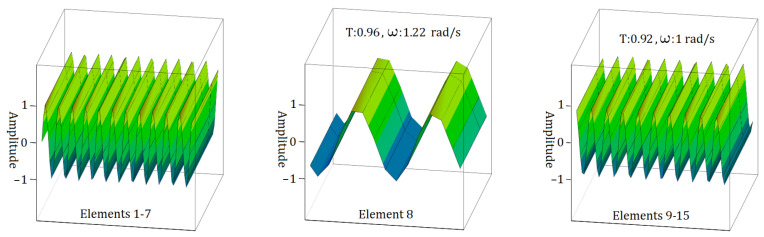
EM wave propagation behaviour in sample S2 with a single element in the transition zone for the case of n form.

Figure 10 and Figure 11 visually represent the intricate behaviour of electromagnetic waves as they propagate through the sample S2. As shown in Figure 10, the v form of the S2 sample transition zone exhibits the lowest electromagnetic wave propagation frequency value, ω = 0.707 rad/s, as determined through the analysis of the Element 8 state. A comparison of these findings with those from analyses of other element numbers reveals a reduction in electromagnetic frequency by half. Furthermore, the transmission ratios (T) gradually decline, initially to 0.89 and subsequently to 0.79.

As can be seen from Figure 11, the analysis indicates that when the transition zone of the S2 sample is n form, the highest observed frequency for electromagnetic wave propagation in the Element 8 state is ω = 1.22 rad/s. Furthermore, analyses of the other elements consistently yield an electromagnetic wave propagation frequency of ω = 1.22 rad/s. Concurrently, the transmission ratios (T) exhibit a slight decrease, initially declining to 0.96 and subsequently to 0.92.

A detailed examination of Figure 7 and Figure 11 reveals a noticeable decrease in the energy of the electromagnetic wave during its propagation. Specifically, when considering the wave propagation in the ‘v’ form of the S1 and S2 samples, this decrease in energy is significant. In contrast, when analysing the ‘I’ form of the S1 sample and the ‘n’ form of the S2 sample, there is no significant change in the energy of the electromagnetic wave during its travel.

## 4. Conclusions

In this study, two samples, S1 and S2, were fabricated using rotary inertia friction welding and a universal lathe. The primary objective of the study was to investigate the electromagnetic wave propagation behaviour in these samples. Specifically, the study focused on modelling the heat-affected zones (HAZs) of the produced samples in various forms and analysing the electromagnetic wave propagation behaviour in these regions. What distinguishes this study from others in the literature is its exploration of the EM wave propagation behaviour not only in the separate segments where the samples were formed but also in the transition zones of these samples. This study investigates the behaviours of electromagnetic wave propagation in samples divided into three distinct components: Part I, the heat-affected zone (HAZ), and Part II. Utilising the finite element method, the analysis breaks these components down into individual elements, acknowledging potential variations in the material property parameters of each element. The electromagnetic wave propagation behaviours of these elements are then examined accordingly. While Parts I and II are modelled homogeneously, the region between them—the HAZ—displays a non-homogeneous distribution of material property parameters. As a result, this modelling approach specifically addresses this complexity. Upon reviewing alternative studies in the literature, it becomes clear that most focus on the thermal or mechanical characteristics of HAZ in samples produced via friction welding. This study, however, stands out by concentrating on the examination of electromagnetic wave propagation behaviours in these samples, particularly within their HAZ.

The finite element method was chosen for analysis due to its low error levels, typically around a maximum of 5%, and the ease of applying the solution. This study introduces a novel perspective to classical research by examining the EM wave propagation behaviour in photonic materials. Furthermore, the methodological approach using the finite element method holds the potential for application to diverse problem-solving scenarios. For example, it can be leveraged to ascertain the electromagnetic wave propagation properties influenced by the formation of tin whiskers in electronic circuit elements experiencing thermal cycling as well as to investigate the electromagnetic wave propagation properties in environments where material properties may undergo change at elevated temperatures, such as tanks in nuclear power plants. The analyses reveal notably low reflection rates for the I form of the transition zone in the S1 sample and the n form of the S2 sample. This results in high transmission rates, approximately T: 0.95. These findings suggest that materials with these properties could be effectively utilised in stealth technology.

## Figures and Tables

**Table 1:** Material property parameters of S1 sample related to electromagnetic wave propagation.

S1		Rod I					Transition Zone					Rod II				
k:1		El.1	El.2	El.3	El.4	El.5	El.6	El.7	El.8	El.9	El.10	El.11	El.12	El.13	El.14	El.15
**5 Element**	I	1					1.166	1.333	1.5	1.666	1.833	2				
	v	1					0.833	0.666	0.5	1	1.5	2				
	n	1					1.5	2	2.5	2.333	2.166	2				
**3 Element**	I	1					1	1.25	1.5	1.75	2	2				
	v	1					1	0.75	0.5	1.25	2	2				
	n	1					1	1.75	2.5	2.25	2	2				
**1 Element**	I	1					1	1	1.5	2	2	2				
	v	1					1	1	0.5	2	2	2				
	n	1					1	1	2.5	2	2	2				

**Table 2:** Material property parameters of S2 sample related to electromagnetic wave propagation.

S2		Rod I					Transition Zone					Rod II				
k:1		El.1	El.2	El.3	El.4	El.5	El.6	El.7	El.8	El.9	El.10	El.11	El.12	El.13	El.14	El.15
**5 Element**	v	1					0.833	0.666	0.5	0.666	0.833	1				
	n	1					1.166	1.333	1.5	1.333	1.166	1				
**3 Element**	v	1					1	0.75	0.5	1.75	1	1				
	n	1					1	1.25	1.5	1.25	1	1				
**1 Element**	v	1					1	1	0.5	1	1	1				
	n	1					1	1	1.5	1	1	1				

**Table 3:** Error analysis results related to the S1 sample.

S1		Rod I					Transition Zone					Rod II				
							Error %									
k:1		El.1	El.2	El.3	El.4	El.5	El.6	El.7	El.8	El.9	El.10	El.11	El.12	El.13	El.14	El.15
**1 Element**	I	0					0	0	0.024	0	0	0				
	v	0					0	0	0.028	0	0	0				
	n	0					0	0	0.031	0	0	0				
**3 Element**	I	0					3.444	3.444	0.024	2.315	2.315	0				
	v	0					1.670	1.670	3.507	4.396	4.396	0				
	n	0					3.037	3.037	0.711	2.333	0.574	0.042				
**5 Element**	I	0					1.576	1.845	1.845	1.139	2.697	0.424				
	v	0					0.219	0.228	0.228	0.240	0.100	0.014				
	n	0.1					0.232	0.353	0.363	0.363	0.336	0.353				

**Table 4:** Error analysis results related to the S2 sample.

S2		Rod I					Transition Zone					Rod II				
									Error %							
k:1		El.1	El.2	El.3	El.4	El.5	El.6	El.7	El.8	El.9	El.10	El.11	El.12	El.13	El.14	El.15
**1 Element**	v	0					0	0	0.028	0	0	0				
	n	0					0	0	0.024	0	0	0				
**3 Element**	v	0					0	0.051	0.028	0.051	0	0				
	n	0					0	0	0.024	0.022	0	0				
**5 Element**	v	0					0	0	0.233	0.440	0.410	0				
	n	0					0	0.323	0.318	0.323	0.701	0				

## Data Availability

Data supporting the results reported in this paper may be obtained from the authors upon reasonable request.

## References

[1] Cornwell D. (2016). Space-based laser communications break threshold. Opt. Photonics News.

[2] Araki K., Arimoto Y., Shikatani M., Toyoda M., Toyoshima M., Takahashi T., Kanda S., Shiratama K. Performance evaluation of laser communication equipment onboard the ETS-VI satellite. Proceedings of the SPIE (Proceedings SPIE—The International Society for Optical Engineering).

[3] Karakilinc O.O., Dinleyici M.S. (2015). Design of dual-mode dual-band photonic crystal bandpass filters for terahertz communication applications. Microw. Opt. Technol. Lett..

[4] Guenneau S., Geuzaine C., Nicolet A., Movchan A.B., Zolla F. (2004). Low frequency electromagnetic waves in periodic structure. Int. J. Appl. Electromagn. Mech..

[5] Basmaci A.N. Electromagnetic frequency characteristics of periodically graded wires. Proceedings of the 2019 PhotonIcs & Electromagnetics Research Symposium (PIERS).

[6] El Haddad A. (2016). Exact solution for the electromagnetic wave propagation in a photonic band gaps material with sinusoidal periodicity of dielectric permittivity. Optik.

[7] Shi J.-M., Yuan Z.-C. (2015). Tunable filter using defect in one-dimensional plasma photonic crystals. Int. J. Appl. Electromagn. Mech..

[8] Fan W., Dong L. (2010). Tunable one-dimensional plasma photonic crystals in dielectric barrier discharge. Phys. Plasmas.

[9] Fathallah W., Sakli H., Aguili T. (2014). Electromagnetic wave propagation in anisotropic metamaterial waveguides. Int. J. Numer. Model..

[10] Chen J., Dai Y., Yan L., Zhao H. (2018). Steady bound electromagnetic eigenstate arises in a homogeneous isotropic linear metamaterial with zero-real-part-of-impedance and nonzero-imaginary-part-of-wave-vector. Opt. Commun..

[11] Hirani R.R., Pathak S.K., Shah S.N., Sharma D.K. (2018). Dispersion characteristics of dielectric tube waveguide loaded with plasma for leaky wave antenna application. Int. J. Electron. Commun..

[12] Qin L., Du W., Cipiccia S., Bodey A.J., Rau C., Mi J. (2024). Synchrotron X-ray operando study and multiphysics modelling of the solidification dynamics of intermetallic phases under electromagnetic pulses. Acta Mater..

[13] Kubrusly A.C., Tovar P., von der Weid J.P., Dixon S. (2021). Mode conversion of SH guided waves with symmetry inversion in plates. Ultrasonics.

[14] Tietze S., Lindner G. (2019). Visualization of the interaction of guided acoustic waves with water by light refractive vibrometry. Ultrasonics.

[15] Wang Y., Gao T., Liu D., Sun H., Miao B., Qing X. (2020). Propagation characteristics of ultrasonic weld-guided waves in Friction stir welding joint of same materials. Ultrasonics.

[16] Aspelmeyer M., Kippenberg T.J., Marquardt F. (2014). Cavity optomechanics. Rev. Mod. Phys..

[17] Wright D.W., Cobbold R.S.C., Yu A.C.H. Ultrasound wave propagation in time-varying phononic crystals. Proceedings of the 2008 IEEE Ultrasonics Symposium.

[18] Fomenko S.I., Golub M.V., Zhang C., Bui T.Q., Wang Y.-S. (2014). In-plane elastic wave propagation and band-gaps in layered functionally graded phononic crystals. Int. J. Solids Struct..

[19] Ponge M.-F., Croenne C. (2016). Control of elastic wave propagation in one-dimensional piezomagnetic phononic crystals. J. Acoust. Soc. Am..

[20] Rayleigh L. (2009). XXVI. On the remarkable phenomenon of crystalline reflexion described by Prof. Stokes. Lond. Edinb. Dublin Philos. Mag. J. Sci..

[21] Chung K.H., Kato T., Mito S., Takagi H., Inoue M. (2010). Fabrication and characteristics of one-dimensional magnetophotonic crystals for magneto-optic spatial light phase modulators. J. Appl. Phys..

[22] Basmaci A.N. (2020). Characteristics of electromagnetic wave propagation in a segmented photonic waveguide. J. Optoelectron. Adv. Mater..

[23] Ranjbar M., Bahari A. (2016). Investigation of third order nonlinearity in propagation of cylindrical waves in homogeneous nonlinear media. Opt. Commun..

[24] Xiao Y., Maywar D.N., Agrawal G.P. (2014). Reflection and transmission of electromagnetic waves at a temporal boundary. Opt. Commun..

[25] Kaya N., Delihacioglu K. (2014). Reflection and transmission coefficients from chiral nihility slab. J. Optoelectron. Adv. Mater..

[26] Qu Z., Wang Y., Wang W., Yu D. (2021). Robust magnetic and electromagnetic wave absorption performance of reduced graphene oxide loaded magnetic meta nanoparticle composites. Adv. Powder Technol..

[27] Fang F., Jing W.Q., Yang W. (2018). High performance electrospinning fiberous membranes for infrared stealth camouflage. Infrared Phys. Technol..

[28] Zohuri B. (2020). Radar Energy Warfare and the Challenges of Stealth Technology.

[29] Pozar D.M. (2012). Microwave Engineering.

[30] Cheng D.K. (2013). Field and Wave Electromagnetics.

[31] Monk P. (2003). Finite Element Methods for Maxwell’s Equations.

[32] Reddy J.N. (2004). An Introduction to Nonlinear Finite Element Analysis.

[33] Chen J., Wu C., Ying J. (2023). Application of extended finite element method for studying crack propagation of welded strip steel in the cold rolling process. Materials.

[34] Qu J., Xue H., Li Y., Chai Y. (2022). An enriched finite element method with appropriate interpolation cover functions for transient wave propagation dynamic problems. Mathematics.

[35] Raffaele D., Rustighi E., Waters T. (2023). Semi-Analytical finite-element analysis for free and forced wave propagation using COMSOL and LiveLink for Matlab. Vibration.

[36] Zhang Q., Zhao M., Huang J., Du X., Zhang G. (2023). A 2.5D finite element method combined with zigzag-paraxial boundary for long tunnel under obliquely incident seismic wave. Appl. Sci..

[37] Melquiades de Dios-Leyva M., Márquez-González A., Duque C.A. (2023). Exploring photonic crystals: Band structure and topological interface states. Condens. Matter.

[38] Khidhir G.I., Baban S.A. (2019). Efficiency of dissimilar friction welded 1045 medium carbon steel and 316L austenitic stainless steel joints. J. Mater. Res. Technol..

[39] Bussu G., Irving P.E. (2003). The role of residual stress and heat affected zone properties on fatigue crack propagation in friction stir welded 2024-T351 aluminium joints. Int. J. Fatigue.

[40] Moat R., Karadge M., Preuss M., Bray S., Rawson M. (2008). Phase transformations across high strength dissimilar steel inertia friction weld. J. Mater. Process. Technol..

[41] Basmaci A.N., Filiz S., Şahin M. (2020). Experimental analysis of welded rods with a functionally graded material approach. Appl. Sci..

[42] Monorchio A., Bretones A.R., Mittra R., Giuliano M., Martin R.G. (2004). A hybrid time-domain technique that combines the finite element, finite difference and method of moment techniques to solve complex electromagnetic problems. IEEE Trans. Antennas Propag..

[43] Kucharski A.A. (2018). The FIT-MoM hybrid method for analysis of electromagnetic scattering by dielectric bodies of revolution. IEEE Trans. Antennas Propag..

[44] Azadifar M., Dehkhoda P., Sadeghi A.H.H., Moini R. (2014). A hybrid FDTD-MoM technique for susceptibility evoluation of a transmission line inside a perforated enclosure. IEEE Trans. Antennas Propag..

[45] Dorum C., Lademo O.-G., Myhr O.R., Berstad T., Hopperstad O.S. (2010). Finite element analysis of plastic failure in heat-affected zone of welded aluminium connections. Comput. Struct..

[46] Shabgard M., Oliaei S.N.B., Seyedzavvar M., Najadebrahimi A. (2011). Experimental investigation and 3D finite element prediction of the white layer thickness, heat affected zone, and surface roughness in EDM process. J. Mech. Sci. Technol..

